# Human Skin Permeation Enhancement Using PLGA Nanoparticles Is Mediated by Local pH Changes

**DOI:** 10.3390/pharmaceutics13101608

**Published:** 2021-10-03

**Authors:** Javiana Luengo, Marc Schneider, Anna M. Schneider, Claus-Michael Lehr, Ulrich F. Schaefer

**Affiliations:** 1 Department of Pharmacy, Biopharmaceutics and Pharmaceutical Technology, Saarland University, 66123 Saarbrücken, Germany; jluengo@udec.cl (J.L.); a_henning@gmx.net (A.M.S.); ufs@mx.uni-saarland.de (U.F.S.); 2 Departamento de Farmacia, Facultad de Farmacia, Universidad de Concepción, Concepción 4070043, Chile; 3 Helmholtz Institute for Pharmaceutical Research Saarland (HIPS), Helmholtz Center for Infection Research (HZI), 66123 Saarbrücken, Germany

**Keywords:** PLGA, polymer nanoparticles, pH effects, human skin, permeation, penetration

## Abstract

The steady improvement and optimization of transdermal permeation is a constant and challenging pharmaceutical task. In this study the influence of poly(lactide-co-glycolide) (PLGA) nanoparticles on the dermal permeation of the anti-inflammatory drug flufenamic acid (FFA) was investigated. For this aim, different vehicles under non-buffered and buffered conditions and different skin models (human heat separated epidermis and reconstructed human epidermis equivalents) were tested. Permeation experiments were performed using static Franz diffusion cells under infinite dosing conditions. Already the presence of drug-free nanoparticles increased drug permeation across the skin. Drug permeation was even enhanced when applying drug-loaded nanoparticles. In contrast, buffered vehicles with different pH values (pH 5.4–7.4) revealed the influence of the pH on the permeation of FFA. The change of the surrounding pH of the biodegradable nanoparticulate system was demonstrated and visualized using pH-sensitive fluorescent probes. While a potential contribution of hair follicles could be ruled out, our data suggest that the enhanced permeation of FFA through human skin in the presence of PLGA nanoparticles is mediated by a locally decreased pH during hydrolytic degradation of this polymer. This hypothesis is supported by the observation that skin permeation of the weak base caffeine was not affected.

## 1. Introduction

The skin, due to the structure and composition of the stratum corneum, provides a highly impermeable barrier of the human body against the environment. However, the same features limit the dermal absorption of active compounds. Therefore, several strategies to improve the transdermal delivery of active pharmaceutical ingredients have been investigated e.g., by means of physical/mechanical enhancement, by supersaturated formulations, ionto- and sonophoresis, electroporation, microneedles, chemical penetration enhancers and nanosized drug delivery systems [1,2]. Nowadays, nanosized drug delivery systems have gained a broad attention for use in dermal/transdermal delivery due to the possibility of delivering the active compound in a controlled manner to or across skin [3,4,5,6]. During the last decades, polymer-based biodegradable nanoparticles in particular have attracted considerable attention as drug delivery vehicles for which both enhanced penetration [7,8] as well as prolonged release [9] have been reported. In comparison to other drugs in solution, the permeation of the anti-inflammatory drug flufenamic acid encapsulated into poly(lactide-co-glycolide) nanoparticles (PLGA-NP) through human skin was improved [7]. Moreover, the same effect occurred by simply adding drug-free nanoparticles (PLGA-DF-NP) to FFA in solution [7]. In contrast to reports of Riviere et al. concerning fullerene nanoparticles [10] we could not show any absorption for PLGA-NP by the skin using multiphoton microscopy [11]. The nanoparticles accumulated within dermatoglyphs and did not enter the Stratum corneum. Consequently, enhanced follicular transport [12,13] could potentially be responsible for this. Therefore, follicle-free reconstructed skin equivalents were used to address their influence. Furthermore, the known hydrolytic degradation of PLGA into lactic and glycolic acid and the accompanying pH change should also be investigated [14]. In this context, potential pH effects of the nanoparticles on skin permeation were studied at buffered and non-buffered conditions. Furthermore, a pH-sensitive fluorescent dye was used to monitor the pH within the microenvironment of PLGA particles. To prove that the change of ionization of flufenamic acid (log P = 4.88 [15], pKa = 3.9 [16], MW 281.1 [17]) is due to the change of pH, an additional experiment with caffeine (log P = −0.08, pKa 14.0 [18], MW 194.2 [17]) was performed.

## 2. Materials and Methods

### 2.1. Materials

Materials used were: Hydroxyethyl cellulose (HEC) Natrosol^®^ 250 M (Aqualon, Hercules Inc., Wilmington, DE, USA), Flufenamic acid, modification II (Kali-Chemie Pharma, Hannover, Germany), Poly(D,L-lactic-co-glycolic acid) (lactic to glycolic acid ratio 50:50) and a Mw distribution of 40,000–75,000 Da (Sigma Chemical Co., St. Louis, MO, USA), caffeine (Sigma Chemical Co., St. Louis, MO, USA), Polyvinyl alcohol (PVA) Mowiol^®^ 4-88 (Kuraray Specialities Europe GmbH, Frankfurt, Germany). Ringer solution, McIlvaine citric acid phosphate buffer at different pH values, Phtalate buffer (pH 5.2), Phosphate buffer (pH 6) were obtained from Merck, Darmstadt, Germany as well as phosphoric acid and acetonitrile, n-octanol, propanol and potassium phosphate. Sodium hydroxide solution (0.1 M), Hydrochloric acid (0.1 M) was bought from Grüssing GmbH, Filsum, Germany. Methanol Chromasolv^®^ for HPLC was from Sigma-Aldrich GmbH, Seelze, Germany. Ethyl acetate came from Fluka Chemie GmbH, Bucks, Switzerland. Cellulose membrane MWCO 12,000–14,000 Da for permeation experiments were acquired from Medicell International Ltd., London, LX, USA. Arabic gum (Caeser & Lorentz, Hilden, Germany), Gelatine A (Dt. Gelatine Fabriken, Eberbach, Germany), LysoSensor™ Green DND-189 (Invitrogen GmbH, Karlsruhe, Germany) were, as all other compounds, used as obtained from the suppliers.

### 2.2. Equipment

The HPLC system used for drug analysis was a ChromeleonTM Version 6.5 SP2, build 968 with a P580 Pump, an ASI-100 automated sample injector, a STH 585 Column oven and an UVD 170S Detector from DionexSoftro GmbH (Germering, Germany). Franz diffusion cell used was static (type 4G-01-00-15) from PermeGear (Riegelsville, PA, USA). NP preparation made use of a high-speed homogenizer Ultra-Turrax^®^ T25 (Jahnke & Kunkel GmbH & Co. KG, Staufen, Germany) and Rotavapor^®^ R-205 (Büchi, Flawil, Switzerland). NP size characterization was performed with a Zetasizer^®^ Nano (Malvern Instruments GmbH, Herrenberg, Germany) and a Confocal laser scanning microscope (CLSM) MRC 1024 (BioRad/Carl Zeiss AG, Jena, Germany) and an atomic force microscopy (AFM) Dimension system, Digital Instruments (today Bruker Corporation, Billerica, MA, USA) was used for visualization of the particles and the surrounding.

### 2.3. Nanoparticles Preparation and Characterization

PLGA loaded (FFA NP) and drug-free nanoparticles (DF-NP) were prepared by solvent extraction method as described before [7]. In brief, thirty milligrams of FFA were dissolved in 600 mg PLGA in 20 mL ethyl acetate. The organic phase was then added dropwise into the same volume of an aqueous phase (20 mL), containing 1% of polyvinyl alcohol as stabilizer, under stirring. The resulting *o/w* emulsion was homogenized with a high-speed homogenizer at 13,500 rpm for 10 min. The amount of water was increased to 200 mL to precipitate the polymer/drug phase. Subsequently, the organic solvent was removed using a rotating evaporator. Drug-free particles were prepared in the same way.

To also evaluate a potential size effect of the NP two different sizes were produced (270 nm and 470 nm) by varying the homogenizing speed.

The PLGA NP were characterized using photon correlation spectroscopy (PCS) using a Zetasizer Nano (Malvern, UK) and atomic force microscopy (AFM) using tapping mode at a Dimension system, Digital Instruments (Bruker) [7]. The particles used had an entrapment efficiency of 63.6% *w/w* FFA and PDI for all preparations were lower 0.1 indicating a narrow size distribution. This was confirmed by AFM pictures also revealing the homogenous and smooth surface structure of the particles. Those particles were already used in previously published work [7] and therefore, will not be described in detail or discussed here again.

### 2.4. Microparticles from PLGA and Arabic Gum/Gelatine A

PLGA microparticles were obtained by emulsion technique based on the same composition as the nanoparticles but with lower homogenization speed (8000 rpm for 2 min).

The preparation of the arabic gum/gelatine. A microparticles as reference particles was done by dissolving gelatine A in distilled water. Then several drops of n-octanol as well as the dissolved arabic gum were added. Afterwards, distilled water at 50 °C was added and the pH was adjusted to 4 using acetic acid. After overnight cooling, the particles were re-dispersed in propanol for post-curing and dried.

### 2.5. Non-Buffered Gel Preparations

Hydroxyethyl cellulose (HEC) hydrogels of flufenamic acid (FFA HG) were prepared from flufenamic acid dissolved in water. To accelerate dissolution a minimal amount of sodium hydroxide solution was added (later neutralized adding hydrochloric acid) under vigorous stirring. Afterwards, HEC was added in a proportion equivalent to 1.5% (*w/w*) and stirred overnight until the polymer was completely swollen. The absence of crystals was confirmed by microscopic inspection. For the preparation of non-buffered gels containing the NP, the original NP suspension was concentrated. A total 20 mL of freshly prepared nanoparticle suspensions was centrifuged at 2468× *g* for 5 min, 15 mL of the supernatant was discarded and the particles re-suspended. Subsequently, their drug content was determined and adjusted by dilution with distilled water to the required concentration.

Flufenamic acid nanoparticle hydrogel (FFA NP HG) was prepared using a HEC gel (3% *w/w*) which was mixed in a 1:1 ratio with a concentrated aqueous suspension of the nanoparticles to obtain the same concentration as used for the drug containing gel (FFA HG).

A hydrogel with incorporated drug-free nanoparticles (FFA HG + DF-NP) was prepared using flufenamic acid and HEC concentration with doubled concentrations, prepared as described for FFA HG and mixed with a concentrated drug-free nanoparticle suspension in a proportion 1:1.

To the nanoparticle-containing gels, equal amounts of NaOH and HCl solutions as in FFA HG were added. The presence and integrity of the spherical nanoparticles in the gel was confirmed by AFM after deposition and drying on a flat surface as described earlier [7].

Permeation experiments were carried out in quadruplicate for FFA HG, FFA NP HG and FFA HG + DF-NP, each with a drug concentration of 0.125 ± 0.006 mg/g.

### 2.6. Determination of the Saturation Concentration of Flufenamic Acid in Different Solutions

The analysis of the saturation concentration in different vehicles was performed as follows: 500 mg of flufenamic acid were added into a volumetric flask (500 mL) and Soerensen phosphate buffer (pH 6.0) and McIlvaine buffer solutions at pH values between 3.4 and 7.4 (for composition, please refer to [19]). All the different mixtures were stirred at 500 rpm for 48 h at 32 ± 1 °C. Afterwards they were left to sediment for 12 h. Three samples, of 10 mL each, were withdrawn from each flask and filtered through 0.2 µm-PTFE filter (OptiFlow) at 32 ± 1 °C. The first 7.5 mL were not used for analysis, and the last 2.5 mL were transferred to a separate flask. One milliliter of the filtered solution was diluted to 100 mL with NaOH 0.05 M and analyzed by HPLC.

### 2.7. Buffered Gels Preparation with Soerensen Buffer

Buffered gels were prepared as described above using HEC and Soerensen phosphate buffer pH 6.0 instead of water.

Permeation experiments were carried out at least in triplicate using FFA HG, FFA NP HG and FFA HG + DF-NP, each with a drug concentration of (0.125 ± 0.006) mg/g. The pH of the final preparations was in accordance with the respective buffer solution (±0.1 unit).

### 2.8. Flufenamic Acid Solutions and NP Suspensions

#### Non-Buffered Formulations

A concentrated solution of flufenamic acid (98.85 µg/mL) was prepared in NaOH 0.04 M.

Non-buffered solutions of flufenamic acid (FFA sol), non-buffered FFA-loaded nanoparticle suspension (FFA NP), and non-buffered solutions with drug-free nanoparticles (FFA + DF-NP) were prepared mixing the components from Table 1.

Buffered formulations with various MCIlvaine buffers:

One hundred milliliters of McIlvaine concentrated buffers were prepared by adjusting the composition from Table 2.

Flufenamic acid solution (FFA sol), loaded nanoparticles (FFA NP) and buffered formulations containing drug free nanoparticles were prepared as follows: Buffer solution and water were mixed with the drug and stirred overnight to dissolve the drug. Afterwards, concentrated nanoparticle suspension was added to the mixture (when corresponds). See composition in Table 3.

To determine the drug concentration, the drug was extracted from all preparations and diluted with NaOH 0.05 M and the samples were analyzed by HPLC. The concentration of all liquid formulations was in the range of 37.6 ± 1.9 µg/mL.

### 2.9. Caffeine Solution and NP Suspension

Caffeine solution was prepared by dissolving 100 mg caffeine in 100 mL of water under stirring overnight resulting in a concentration of 1 mg/mL (0.1%).

Caffeine solution with drug free PLGA nanoparticles was prepared by mixing a 0.2% caffeine solution with a suspension of drug free PLGA nanoparticles in a ratio of 1:1 (*v/v*).

### 2.10. Skin Preparation

Excised human skin from Caucasian female patients, who had undergone abdominal plastic surgery, was used. The procedure was approved by the Ethical Committee of the Aerztekammer des Saarlandes, Saarbrücken, Germany (Code 204/08, 22 December 2008). Adequate health and no medical history of dermatological disease were required. After excision, subcutaneous fatty tissue was separated from whole dermis using skin pieces 10 × 10 cm by means of a scalpel. The cleaning of the skin surface was done with cotton pads soaked with Ringer buffer, wrapped in aluminum foil, and stored in polyethylene bags at −26 °C until use. Under these conditions no change in permeation characteristics took place within six months [20,21].

Disks with a diameter of 25 mm were punched out from frozen skin, thawed, cleaned with Ringer solution. These samples were used to prepare the heat separated epidermis sheets then applied during permeation experiments in Franz diffusion cells.

#### 2.10.1. Preparation of Heat Separated Epidermis

The epidermis was separated adding the cleaned skin disk after thawing in a water bath of 60 °C for 90 s. Afterwards, the skin was transferred, dermal side down, on a filter paper. Forceps were used to peel off the epidermal layer from the skin. Before use for permeation experiments, the epidermal membrane was pre-hydrated for 1 h.

#### 2.10.2. Reconstructed Human Epidermis Equivalents

The reconstructed human epidermis model SkinEthic RHE/L/17 (4.0 cm^2^) was provided by Laboratoire SkinEthic (Nice, France) and is described as an epidermal model based on air-lifted culture of normal human keratinocytes for 17 days in chemically defined medium on inert polycarbonate filters [22,23]. The morphology of SkinEthic RHE/L/17 is highly similar to human tissue showing structures like stratum corneum, stratum granulosum and stratum spinosum. More details for the lipid composition and biochemical markers of the reconstructed skin can be found in Netzlaff et al. [22].

For permeation experiments SkinEthic RHE/L/17) was punched out and used parallel to heat separated epidermis.

#### 2.10.3. Permeation Experiments

The permeation experiments were carried out using static Franz diffusion cells (FD-C) with either heat-separated epidermis or reconstructed skin (SkinEthic). The skin membrane was mounted on a cellulose membrane disk. The membrane was located between the donor and receptor compartment of the FD-C. The donor phase was 0.75 mL gel or 1 mL of drug solution or NP preparation for infinite dose experiments were used. As acceptor served 12.1 mL of Soerensen phosphate buffer (pH 7.4). The donor compartment was closed with sheets of aluminum foil and the system was kept at constant temperature (32 ± 1) °C in a water bath. The acceptor was agitated at 500 rpm using a magnetic bar. At predetermined time intervals, samples of 0.3 mL were withdrawn from the acceptor compartment and replaced immediately with fresh buffer solution. Samples were collected over 30 h and analyzed by HPLC. Integrity was checked visually by a magnifying glass after mounting the skin specimen on the Franz diffusion cell.

For permeation experiments with caffeine phosphate buffered saline (PBS) was used as acceptor medium.

#### 2.10.4. HPLC Methods

Flufenamic acid:

All samples were analyzed using the following HPLC conditions: Column LiChrospher 100 RP-18, 5 μm, 125 × 4 mm (Merck, Darmstadt, D); Mobile Phase: McIlvaine buffer pH 2.2: Methanol (20:80); Flow rate: 1.2 mL/min; Wavelength: 284 nm; Injection volume: 20 μL; Retention time: (3.5 ± 0.2) min, lower limit of detection 50 ng/mL. This method has been previously validated by Wagner et al. [24]. This method has been previously validated by Wagner et al. [24].

Caffeine:

All samples were analyzed using the following HPLC conditions: Column LiChrospher 100 RP-18, 5 μm, 125 × 4 mm (Merck, Darmstadt, D); Mobile Phase: buffer pH 2.2 (composed of 1.16 mL phosphoric acid, 4.08 g potassium phosphate, water ad 1000 mL): Acetonitrile (10:90 *v/v*); flow rate: 1.2 mL/min; Wavelength: 262 nm; Injection volume: 50 μL; Retention time: (3.1 ± 0.2) min. Lower limit of quantification: 30 ng/mL.

### 2.11. CLSM-Measurements

For determination of the local pH of the particle samples the pH of the particle suspensions was roughly adjusted to pH = 7 using sodium hydroxide. Hereafter, 10 µL suspension was mixed with 10 µL of a 0.1 mM LysoSensor™ solution. The mixture was transferred onto a microscope slide and sealed with a cover slip using nail polish [25]. The fluorescence measurements were performed using the 488 nm line of the argon/krypton laser and a band pass filter (522/35) for each of the particle suspensions. Transmission light images were taken using a conventional light bulb with the red channel of the CLSM (MRC 1024 (BioRad/Carl Zeiss AG, Jena, Germany).

### 2.12. Statistical Evaluation

For statistical evaluation, SigmaStat 3.0.1 was used. ANOVA test was run using “all pairwise comparison procedure” (Holm-Sidak method). All pairwise multiple comparison procedures (Holm-Sidak method) were based on an overall significance level *p* < 0.05.

## 3. Results

### 3.1. Infinite Dose Permeation Experiments Using Unbuffered Hydrogels and Heat Separated Human Epidermis

Infinite dose permeation experiments using unbuffered hydrogels containing dissolved flufenamic acid (FFA), either in presence or absence of drug-free NP or FFA encapsulated into nanoparticles (NPs), were performed in vitro to investigate the influence of NPs on the dermal absorption.

**Figure 1:**
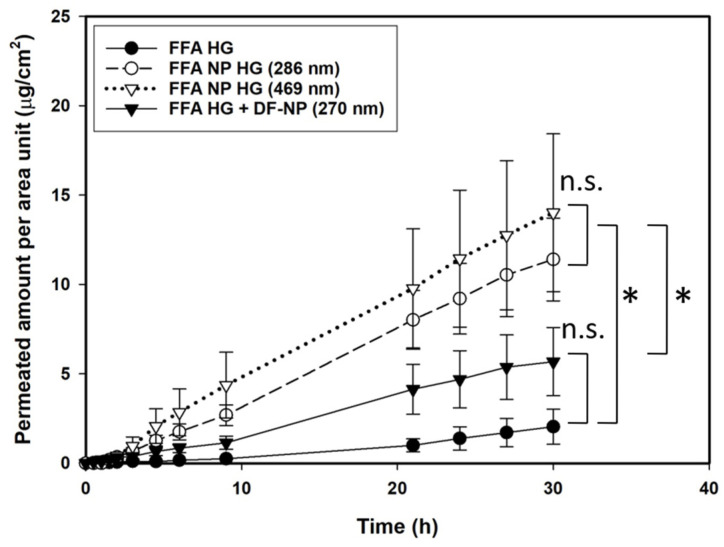
Permeation profiles across heat separated human epidermis of flufenamic acid using infinite dose regime of non-buffered hydrogels containing: dissolved drug (FFA HG), FFA-loaded nanoparticles of different sizes (FFA NP HG), and dissolved drug with drug-free nanoparticles (FFA HG + DF-NP). Concentration 0.125 µg/g. The drug-loaded NP formulations were statistically not different (n.s.) from each other, but both were statistically different (*) from the drug-free formulation and the drug in hydrogel. Statistical evaluation is based on Papp values from 4.5 to 30 h.

As shown in Figure 1 an increase in the permeation of FFA was observed when the drug was encapsulated into PLGA nanoparticles. The drug-loaded formulations were statistically different from the pure drug in hydrogel or co-administered with the drug-free particles in a drug-containing hydrogel. With respect to the influence of the particle size of the particles no differences in skin penetration could be observed (statistically not different). Particles with a mean diameter of 469 nm showed after 5 h the same permeability as the 286 nm particles. When drug-free nanoparticles were co-administered an intermediate effect between dissolved drug and drug-loaded nanoparticles was observed which however, did not reach statistical significance (Holm-Sidak test).

### 3.2. Permeation Experiments Using Hydrogels and Reconstituted Human Epidermis (Skinethic^®^)—Infinite Dose Regime

**Figure 2:**
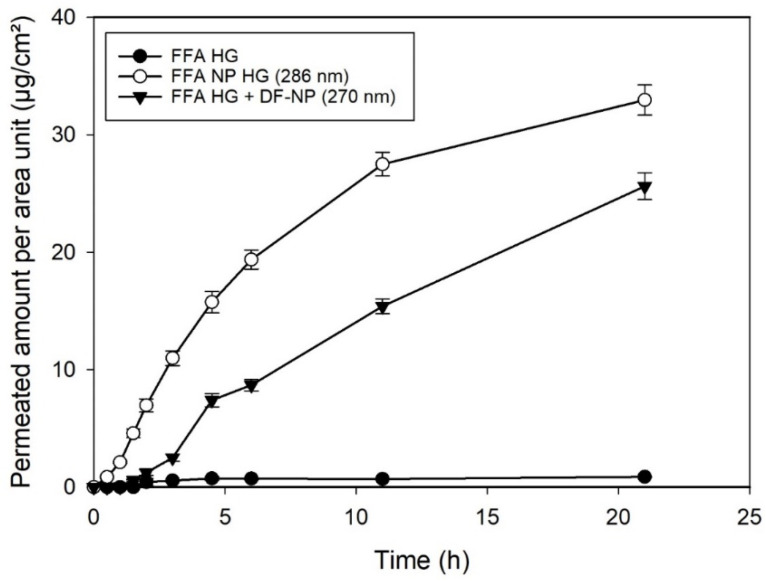
Permeation profiles of reconstructed human epidermis (SkinEthic^®^) using flufenamic acid at infinite dose regime of: dissolved drug in hydrogel (FFA HG), FFA-loaded nanoparticles dispersed in the hydrogel (FFA NP HG), and dissolved drug with drug-free nanoparticles dispersed in the hydrogel (FFA HG + DF-NP). Concentration of FFA 0.125 µg/g.

To elucidate the influence of hair follicles, experiments on reconstructed human epidermal equivalents (SkinEthik^®^) were carried out. The SkinEthic^®^ model is a human reconstructed epidermal model, highly permeable [17,22,26,27] and devoid of hairs and hair follicles. If the enhanced penetration of FFA encapsuled in NPs depends on the accumulation of NPs in the hair follicle minor differences among the formulations would be expected for the reconstituted human epidermis compared to human HSE. But in contrast to these considerations, permeation profiles showed a similar pattern with respect to those observed using human HSE (Figure 2). Drug-loaded NP showed a pronounced permeation across the reconstructed, hair-follicle free skin. These results suggested that the follicular pathway as proposed by Lademann and Toll [13] is not responsible for the increased permeation induced by the nanosized preparations. As particles of these sizes are not expected to penetrate into/through skin this option can be ruled out [11,28]. Surprisingly, DF-NP also show an effect on the drug permeation that was not expected.

In view of the similar penetration enhancement for natural and reconstituted (i.e., follicle-free) epidermis and the effect of the presence of unloaded particles with free drug follicular penetration can be neglected. As an alternative effect, we hypothesized that degradation of the nanoparticles generates a lower pH microenvironment around the particles. This acidification was described for the matrix of microparticles before [14]. Such a shift in pH would favor the non-ionized form of the drug, which is able to penetrate the barrier easier than the ionic form. Degradation of PLGA is well documented [14,29,30,31,32]. We could also in the past visualize by AFM the degradation of the nanoparticles [32]. To substantiate this hypothesis, the following experiments were carried out to elucidate if the pH generated by the degradation of PLGA NPs has any influence on the permeation through the epidermal barrier.

### 3.3. Flufenamic Acid Saturation Concentration and Percentage of Ionized Drug in Solutions of Different pH Values

As for instance concentration gradients will influence the permeation behavior, the saturation concentrations of flufenamic acid at different pH values were determined. Furthermore, the theoretical percentages of ionized drug at every pH were calculated using $\alpha =\frac{1}{1+10^{\mathrm{pka}-\mathrm{pH}}}$ (with α = degree of dissociation, pka = pka value and pH = pH value). The results are depicted in Table 4.

Table 4 shows very clearly that the concentration of saturation of flufenamic acid decreases with lower pH value, however, on the other hand the non-ionized form is enhanced. As the non-ionized form is responsible for passing the Stratum corneum barrier a higher permeation occurs.

### 3.4. Infinite Dose Permeation Studies Using Buffered Hydrogels and Heat Separated Human Epidermis

**Figure 3:**
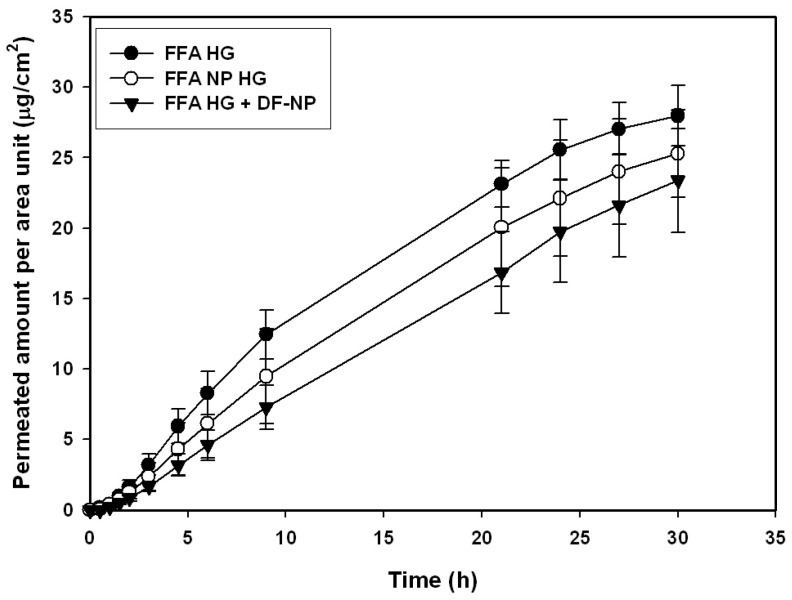
Permeation profiles of human heat separated epidermis of a buffered hydrogel (pH 6.0) using infinite dose regime of flufenamic acid preparations as dissolved drug (FFA HG), FFA-loaded nanoparticles (FFA NP HG), and dissolved drug with drug-free nanoparticles (FFA HG + DF-NP).

In a first approach, the influence of buffering of the hydrogel preparation was investigated. In this way the conditions and the amount of ionized, non-ionized drug was fixed. Permeation experiments with hydrogels buffered with Soerensen phosphate buffer pH 6.0 and the same concentration, as used for previous experiments, were done. Soerensen phosphate buffer was selected due to the compatibility with the gel forming agent (hydroxyethyl cellulose) and the pH at the skin’s surface. When hydrogels buffered to pH 6.0 were examined, differences in permeation diminished in comparison to the unbuffered preparations (Figure 3). Based on these data we concluded that the buffer compensates the effect of the nanoparticles on skin permeation.

### 3.5. Permeation Studies with Non-Buffered and Buffered Solutions in Presence and Absence of Nanoparticles

To rule out the influence of the gel forming agent on the skin permeation FFA solutions with or without unloaded NPs and FFA-loaded nanoparticles in suspension in a non-buffered and buffered form at different pH values were applied. For all these experiments skin from one donor was used.

**Figure 4:**
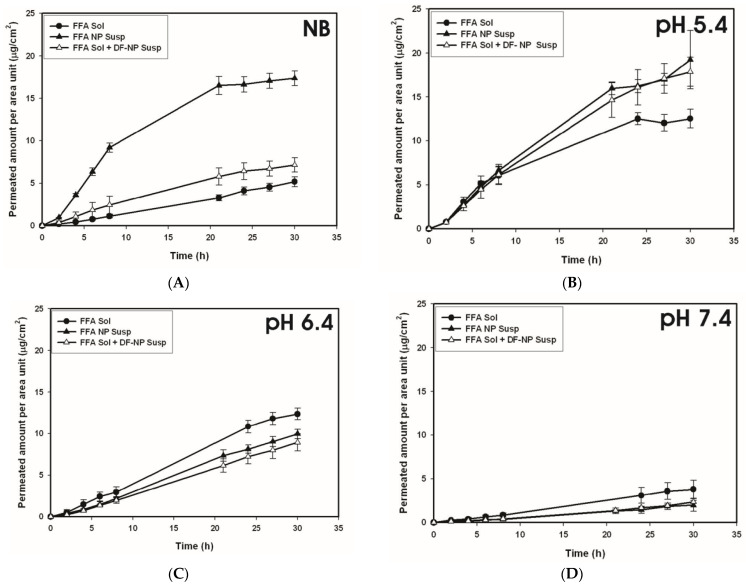
Permeation profiles obtained for human heat separated epidermis using flufenamic acid solution (FFA sol), FFA solution plus drug-free nanoparticles (FFA sol + DF-NP susp) and drug-loaded nanoparticles (FFA NP susp). The formulations were applied as non-buffered preparations (NB, (**A**)) and buffered preparations at different pH values (pH 5.4 (**B**), pH6.4 (**C**), pH 7.4 (**D**)). Concentration of flufenamic acid 37.6 ± 1.9 µg/mL.

For non-buffered systems (see Figure 4A) the highest permeation occurred using FFA-loaded nanoparticles, an intermediate effect was obtained when non-loaded particles were added to FFA in solution and the lowest permeation was found for ‘FFA only’ in solution. These results display the same rank order already established for FFA hydrogel formulations (Figure 1) suggesting that the gel forming agent has no influence on the permeation of FFA through the dermal barrier.

Looking at the buffered systems, it can be clearly seen that the differences diminished between FFA in solution without or with drug free NPs and FFA encapsulated in PLGA-NPs. This is true independent of the tested pH. Moreover, when comparing the preparations at different pH values, a decreased permeation was displayed if pH value rose. This is in accordance with physicochemical models of skin permeation for lipophilic substances that non-ionized forms preferentially permeate the lipophilic pathway of the stratum corneum resulting in an enhanced absorption [34].

### 3.6. Permeation Studies with Caffeine

**Figure 5:**
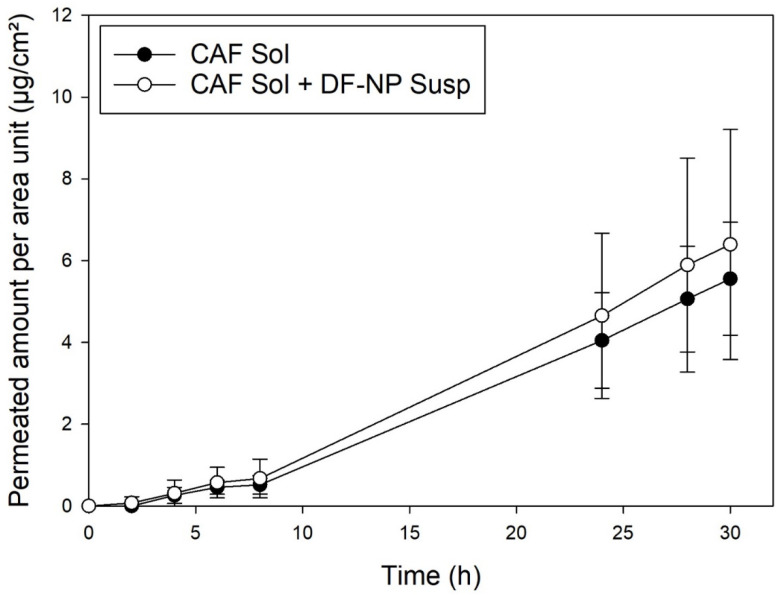
Permeation with heat separated epidermis: caffeine in solution (black, CAF Sol) and caffeine with drug-free PLGA nanoparticles (open symbols, CAF Sol + DF-NP). Caffeine concentration 100 µg/mL.

To further test our pH hypothesis, skin permeation studies with caffeine have been conducted. It was expected that no differences in permeation will be found if aqueous solutions of caffeine without or with drug free NPs are used as the drug should not be affected by potential pH changes. As seen from Figure 5 no difference was displayed between caffeine in solution and caffeine in solution with addition of drug-free PLGA NP. These findings corroborate our hypothesis that change of ionization by an acidic surrounding of the NPs is responsible for the enhancement effects for FFA permeation.

Unfortunately, no PLGA NP loading the hydrophilic caffeine could be prepared using the same preparation approach as used for flufenamic acid (solvent extraction method). The hydrophilic caffeine could not be dissolved in the organic solvent used for PLGA. Thus, an encapsulation of reasonable mount in the PLGA particles was not possible. To achieve such an incorporation the preparation method would have to be modified hampering the conclusion due changing several properties of the particulate system at the time. Thus, a direct comparison of this experimental condition was not possible.

Furthermore, there are reports in literature on the effect of α-hydroxy acids which can reduce the Stratum corneum’s cohesion, allowing to increase permeation of drugs across the skin. For example, Sebastiani et al. [35] have studied the effects of lactic acid on the skin permeation using rabbit skin. Three drugs offering different physicochemical properties were investigated demonstrating that only the passive permeation of ibuprofen, an anionic drug (characteristic present also in flufenamic acid), was affected by the presence of lactic acid. It was found that this led to a clear increase in permeation. Consequently, also if the different pathways remained unchanged, the partitioning from the formulation into the skin was increased [35].

### 3.7. Investigation of the pH in the Surrounding of the Particles’ Surface

**Figure 6:**
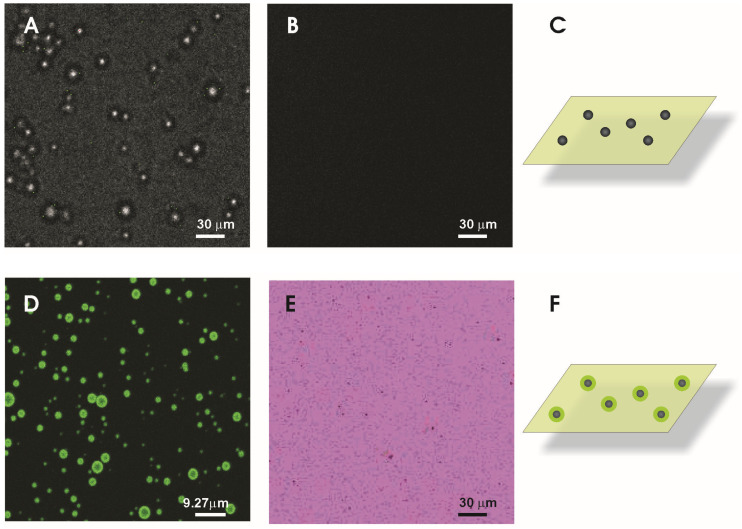
Fluorescence micrographs obtained after dispersing different particle fractions into pH sensor solution. Transmission light image were taken to identify Arabic gum/gelatine A microparticles (**A**) and the corresponding fluorescence image (**B**). PLGA microparticles show green fluorescence localized at the surface of the particles or the close surrounding (**D**). For nanoparticles, fluorescent spots were detected (**E**). For highlighting the particles position the color coding was inverted leading to a pink background with block spots representing the fluorescent nanoparticles. The sketches should illustrate the experimental conditions (**C**,**F**).

To investigate the pH of the micro-surrounding of the particles’ experiments using confocal laser scanning microscopy were performed. As the pH change is linked to the hydrolysis of the NP material it might take place only in the very close (nano-) environment of the particles. However, the optical transfer function of the microscope will limit the resolution. Thus in the micrographs objects smaller than the resolution will be depicted at a dimension equal to the so-called “Airy disc” [36]. For the application of fluorescence-based methods, such as pH-sensitive dyes, the local resolution is therefore also limited and might exceed the extension of the area covered by the particles and the area where the pH change might take place. To overcome these problems regarding the particles, we used PLGA microparticles in the range of several micrometers. This facilitated the investigation of the pH on and close to the surface. As a negative control Arabic gum/gelatine A microparticles were deployed. For pH analysis the dye LysoSensor^®^green enabled us to visualize if different pH values are established even though the precise local determination is not accessible. The LysoSensor^®^ dyes are known as acidotropic compounds. For pH-values above the pKa the dye is unprotonated and the fluorescence is quenched [37]. Therefore, the dye chosen exhibits practically no fluorescence at neutral pH (condition apart from the particles). In acidic environment the molecules are protonated and the fluorescence quenching is relieved; light emission is strongly increased. The images depicted in Figure 6D reveal a strong fluorescence around the PLGA microparticles and indicate that our assumption regarding the particles’ pH is correct. Image 6A reveals the presence of the Arabic gum/gelatine A particles in transmission light whereas in Figure 6B the fluorescence channel is displayed. It can be seen that with those particles no change of the pH and thus no fluorescence is connected. For the PLGA micro- and nanoparticles experiments the background fluorescence was reduced by pre-setting the pH to approximately 7 (The situation is sketched in Figure 6C,F: particles in a slightly fluorescent solvent). The result changes completely exposing the PLGA particles to the same surrounding and measuring conditions as described before. An intense fluorescent signal (Figure 6D,E) was observed. As the location of the fluorescence from the nanoparticles cannot be detected due to the resolution limit of the microscope, PLGA microparticles were used to demonstrate the localization of the fluorescence. The microparticle fluorescence discloses that the light is emitted from/or close to the surface of the particles. The inner particle shows fading fluorescence indicating the absence of fluorescent molecules. For the PLGA nanoparticles the fluorescence is observed as well but due to their size only fluorescent spots are visible which was highlighted by the inverted color coding (Figure 6E). The results clearly indicate the different pH values at the particles’ surface or close environment compared to other particles.

Consequently, a pH-dependent change of drug partitioning into the stratum corneum appears as most plausible explanation for the observed skin permeation enhancement under influence of PLGA nanoparticles. The changed partitioning might result from the local acidic environment around the nanoparticles due to the degradation and a raised fraction of unionized drug. Its lipophilicity and ability to dissociate at physiological skin pH make flufenamic acid a good candidate still, for improved delivery through the skin by such formulations.

## 4. Conclusions

The increased permeation of flufenamic acid through the epidermis from a non-buffered vehicle indicates that PLGA nanoparticles may improve the transdermal delivery of FFA. According to our data, particles of this biodegradable polymer may induce a “corona” of low pH that influences the ionization state of the drug in the micro surroundings of the particles. Therefore, the drug may better penetrate across the skin barrier under such conditions, which may be used advantageously for improved topical delivery of acidic drugs, such as e.g., non-steroidal antirheumatic, without using any additional permeation enhancers.

## Figures and Tables

**Table 1:** Compositions of non-buffered flufenamic acid formulations.

Formulation Component	FFA Sol	FFA NP Suspension	FFA Solution + DF-NP Suspension
FFA concentrated solution	40.464 mL	--	40.464 mL
HCl 0.1 M	1.480 mL	1.480 mL	1.480 mL
NaOH 0.04 M	--	40.464 mL	--
FFA NP concentrated suspension	--	25.536 mL	--
DF-NP concentrated suspension	--		25.536 mL
Deionized water to	100 mL	100 mL	100 mL

**Table 2:** Composition of McIlvaine buffer solutions for buffered flufenamic acid preparations.

Solution Component	pH 5.4	pH 6.4	pH 7.4
Citric acid monohydrate 0.2 M	44.7	31.4	9.8
Disodium phosphate dihydrate 0.4 M	55.3	68.6	90.2

**Table 3:** Composition of buffered flufenamic acid formulations.

Formulation Component	FFA Sol	FFA NP Suspension	FFA Solution + DF-NP Suspension
	FFA sol	FFA NP	FFA + DF-NP
Concentrated buffer	50.000 mL	50.000 mL	50.000 mL
Flufenamic acid	4000 µg		4000 µg
FFA NP concentrated suspension		25.536 mL	
DF-NP concentrated suspension			25.536 mL
Deionized water to	100.000 mL	100.000 mL	100.000 mL

**Table 4:** Saturation concentration and ionized percentage of flufenamic acid in different solvents at 32 °C (mean ± SD).

Solvent		Saturation Concentration (µg/mL)	Ionized Percentage (%)	Non-Ionized Percentage (%)
Water		5.8 ± 0.8	n.d.	n.d.
Soerensen buffer	pH 6.0	29.4 ± 0.4	99.21	0.79
McIlvaine buffer	pH 3.4	1.8 ± 0.2	24.00	76.00
	pH 4.4	5.6 ± 0.5	75.97	24.03
	pH 5.4	43.1 ± 1.8	96.93	3.07
	pH 6.4	383.4 ± 14.2	99.68	0.32
	pH 7.4	2896.1 ± 55.9	99.97	0.03
Soerensen buffer	pH 7.4 *	2059.5 ± 21.6	99.97	0.03

* data were taken from [33]. n.d.: not determined.

## Data Availability

Not Applicable.
